# Early cognitive-linguistic skills as predictors of concurrent and longitudinal poor reader status in Hong Kong Chinese primary school children

**DOI:** 10.1007/s11881-026-00382-1

**Published:** 2026-07-11

**Authors:** Yijun Ruan, Urs Maurer, Catherine McBride

**Affiliations:** 1 Department of Applied Psychology, School of Humanities and Social Sciences, Fuzhou University, Fuzhou, China; 2 Department of Psychology, The Chinese University of Hong Kong, Hong Kong, China; 3 Department of Human Development and Family Science, Purdue University, West Lafayette, Indiana, USA

**Keywords:** Poor readers, Morphological awareness, Orthographic awareness, Rapid automatized naming, Delayed copying, Phonological awareness

## Abstract

The present study investigated the roles of various cognitive-linguistic skills in predicting poor reader status concurrently and longitudinally with a sample of Hong Kong Chinese primary school children. Children from grade 1 to grade 3 (*n* = 358) were measured on tests of Chinese word reading, phonological awareness (PA), morphological awareness (MA), orthographic awareness (OA), rapid automatized naming (RAN), and delayed copying (DC) individually for cross-sectional analyses. Word reading ability was tested annually in a subsample of 109 children from grade 1 (follow-up group) for three years for longitudinal analyses. Children’s word reading performance was utilized to differentiate between poor and typical readers. Results of logistic regression analyses showed that MA and DC were the two significant predictors to distinguish poor readers from typical readers concurrently. Longitudinally, MA, OA, and RAN measured in the first year were the most consistent indicators of poor reader status one and two years later in the follow-up group. PA failed to distinguish Chinese poor readers both concurrently and longitudinally. These results align with the latest IDA dyslexia definition, which emphasizes morphological processing, and extend them by highlighting the critical role of orthographic processing. The findings shed light on the identification and intervention for Chinese poor readers.

## Introduction

Defining reading problems of various sorts across cultures is relatively difficult (e.g., McBride, 2019). Different concepts might be used to represent the same reading problem across contexts. According to the latest definition of dyslexia adopted by International Dyslexia Association (IDA, 2025), dyslexia is a specific learning disability characterized by difficulties in word reading and/or spelling that involve accuracy, speed, or both and vary depending on the orthography. These difficulties occur along a continuum of severity and persist even with instruction that is effective for the individual’s peers (IDA, 2025). Besides phonological processing deficits, the new definition particularly emphasizes morphological processing deficits in dyslexia. The new definition opens up the understanding of dyslexia further, and might be particularly useful to continue to pursue in Chinese, which is a morphosyllabic writing system. Poor word readers refer to individuals who show significant difficulty in word reading. Poor readers might locate at the continuum of dyslexia but they do not receive a professional diagnosis of dyslexia by clinic psychologists. The concept of poor readers and dyslexia have often been mixed and interchangeably used in many previous studies, and the same criteria are often used in different studies to identify poor readers and dyslexia (Liu et al., 2010; Wang et al., 2017). Research in poor readers might shed light on dyslexia, and vice versa. Poor word reading often results in severe problems involving career and mental health (e.g., Lyon et al., 2003). It is essential to identify and intervene with poor readers as early as possible. The present study explored the best correlates of poor reader status concurrently and longitudinally in Hong Kong Chinese primary school children under the context of the latest definition of dyslexia. We not only included phonological and morphological risk indicators that emphasized in the new dyslexia definition, but also extended to the orthographic risk factors. Poor readers in the present study were defined as children whose word reading performance fell in the lowest 25% of all readers in the same grade. This criterion has been adopted in many previous studies as well (e.g., Fong & Ho, 2022; Liu et al., 2010). However, importantly, the spelling dimension is not captured in the poor reader of the present study. Hence, poor reader is a narrower index than the one of dyslexia as encompassing difficulties in word reading and/or spelling as defined by the 2025 IDA definition.

## Characteristics of Chinese script and Hong Kong context

The Chinese writing system is visually complex. The basic writing unit in Chinese is the character, which is composed of different strokes in a square-shaped form. Chinese characters are often described as morphosyllabic because each character simultaneously represents both a syllable and a morpheme (Shu et al., 2006). Most Chinese characters are compound characters comprised of a phonetic radical and a semantic radical. The phonetic radical provides cues for the pronunciation of the character. In contrast, the semantic radical often indicates the meaning of the character in some way (Chung & Ho, 2010). There are many homophones in Chinese. Due to the arbitrary relations between sound and print, learning Chinese characters relies heavily on rote memorizing and repeatedly copying (Chan et al., 2006; McBride-Chang et al., 2011a). According to the psycholinguistic grain size theory, different grain-size units are used to decode words across orthographies (Ziegler & Goswami, 2005). In alphabetic orthographies, phonemes are the main lexical representation, and word reading acquisition relies primarily on the phoneme-grapheme correspondence. In Chinese, phonemes are not explicitly reflected in the script; instead, morphemes become the main lexical representation. Chinese word reading acquisition relies mainly on character-morpheme correspondence. The grain size in Chinese is larger than the grain size in alphabetic orthographies. There are many more orthographic units to learn when the grain size is large than when the grain size is small (Ziegler & Goswami, 2005). The characteristics of different orthographies influence the specific challenges individuals face in learning to use various grain-size units to develop accurate word reading (Catts et al., 2026). Therefore, the risk factors of poor reader in different orthographies might vary, which aligns with the 2025 dyslexia definition.

In Hong Kong, children start to learn to read formally beginning at around age 3. A “look and say” method has commonly been used to teach Chinese traditional characters in Hong Kong. During the teaching, children look at the characters and hear the corresponding pronunciations simultaneously (Ho et al., 2002). Children are required to memorize the connections between the orthographies and pronunciations by rote. Unlike Pinyin used in Mainland China and Zhuyin Fuhao used in Taiwan, there are no similar phonetic coding systems applied in Hong Kong to facilitate the teaching of characters’ pronunciations. Because of these unique circumstances, poor readers in Hong Kong might exhibit different patterns in cognitive-linguistic skills compared to those in other regions (e.g., Ho et al., 2002 vs. Lei et al., 2011).

Several researchers have suggested that the specific features of a given script might influence the manifestations of cognitive deficits related to literacy difficulties (e.g., Bishop & Snowling, 2004). Chinese is a relatively complex script compared to alphabetic scripts. In addition, Chinese and alphabetic orthographies use different grain-size units to acquire word reading. Therefore, the complex Chinese script and the unique Hong Kong context may contribute to distinct markers of poor reader status.

### Cognitive-linguistic skills for Chinese reading acquisition

What are the most important cognitive-linguistic correlates of Chinese reading acquisition? Chinese reading development involves a comprehensive set of cognitive-linguistic skills. Researchers have posited that phonological sensitivity, morphological awareness, orthographic knowledge, and fluency are the four main components of reading across languages (e.g., Xue et al., 2013). A recent meta-analysis examined that reading interventions focused on training fluency, phonological awareness, orthographic awareness, and morphological awareness could effectively improve the literacy skills of Chinese children with and without dyslexia (Ruan et al., 2024). Dyslexia involves broader language foundations and complex interactions among multiple risk factors (Odegard et al., 2025). Therefore, multiple cognitive-linguistic skills should be considered when identifying Chinese poor readers.

Phonological sensitivity is the ability to reflect on and manipulate the phonological structure of spoken words (Wagner et al., 1993). Phonological sensitivity might play an essential role in Chinese reading acquisition because mapping oral Chinese to its graphic representation requires access to the phonology of spoken Chinese (Hu & Catts, 1998). For example, phonological awareness (PA), measured by syllable deletion task, has been revealed to correlate significantly with character reading acquisition in Chinese children (e.g., Yang et al., 2019).

Morphological awareness (MA) refers to the ability to reflect on and manipulate morphemes and morphological structure as well as to distinguish meanings among homophones (McBride-Chang et al., 2003). MA has been found to uniquely contribute to Chinese reading acquisition across children with different reading proficiency levels (e.g., Liu et al., 2017). MA appears to have a particularly prominent role in Chinese reading because of the extensive lexical compounding that is required in Chinese word recognition (Tong et al., 2009). The 2025 IDA definition of dyslexia newly stated morphological processing deficit as one of the common but not universal underlying difficulties in dyslexia besides phonological processing deficit (IDA, 2025). Including MA as one of the predictors of poor reader status could provide evidence for the new definition.

Orthographic knowledge in Chinese includes skills in recognizing the characters’ configuration and structures and in processing radical information within the characters (Ho, 2014). Given the complex features of the Chinese writing system, orthographic knowledge has been found to be essential in Chinese reading development (e.g., Yang et al., 2019). The positional information of radicals is critical in facilitating Chinese learning in early-age children (Leong et al., 2011). Orthographic awareness (OA), which refers to the sensitivity to orthographic regularities in the script, is one aspect of orthographic knowledge (Mo et al., 2018).

Fluency represents the ability to access reading materials quickly and accurately. Rapid automatized naming (RAN) is a primary indicator of fluency. Some researchers have proposed that RAN makes use of both phonological skills and visual skills (Ho et al., 2004) and may also be associated with orthographic processing (Tong et al., 2017). Therefore, it has emerged as a relatively strong correlate of reading acquisition across orthographies, including Chinese (e.g., Xue et al., 2013).

Besides these four core skills, some other skills have also been found to have a salient association with Chinese literacy acquisition. In the present study, we focus on one additional correlate, namely, delayed copying (DC). DC, which taps multiple skills including visual-motor integration, orthographic working memory, and visual-orthographic skills, contributes significantly to Chinese literacy acquisition (Mo et al., 2018). Participants are typically asked to write down an unfamiliar character, string of letters, or any other type of print showing a word that has been displayed for just a few seconds from memory during the activity of DC (Ruan et al., 2023). DC is an independent and underexplored variable beyond the scope of orthographic knowledge. Including DC allows us to examine its different patterns in predicting poor reader status from that of orthographic knowledge. Some researchers have hypothesized that DC might facilitate Chinese reading development by establishing the orthographic representations and long-term motor memory of characters (Tan et al., 2005). Previous studies have found that young Chinese children with different levels of literacy skills showed distinct performances on DC (e.g., Pak et al., 2005).

Overall, studies have consistently revealed that the above cognitive-linguistic skills play important roles in Chinese reading acquisition. While these cognitive-linguistic skills have been shown to contribute to reading acquisition, it is also important to consider how they predict Chinese poor reader status concurrently and longitudinally to facilitate the identification of poor readers. Besides considering phonological and morphological processing factors that specifically noted by the latest IDA dyslexia definition, the present research further probe into the predictive role of orthographic processing in poor reader status. Importantly, both the concurrent and longitudinal roles of these skills in predicting poor reader status are examined in the present study. The results could contribute to the identification of dyslexia from the perspective of Chinese orthography.

## Predictors of concurrent dyslexia/poor reader status in Chinese children

As poor readers and dyslexia are often interchangeably used in many previous studies (e.g., Liu et al., 2010; Wang et al., 2017), the research of predictors of dyslexia should be also reviewed when exploring the correlates of poor reader status. The multiple-deficit model, which suggests that dyslexia is the result of multiple deficits in different skills, is widely accepted by many researchers (e.g., Chung & Ho, 2010). Performances in various cognitive-linguistic skills are reliable factors for distinguishing poor and typical readers concurrently. Weakness in OA, RAN, and MA are the three most dominant deficits in Hong Kong Chinese children with dyslexia (Chung & Ho, 2010). For example, Ho et al. (2002) conducted a study to examine the cognitive profile of Chinese dyslexia, and they found that children with dyslexia performed significantly more poorly on tasks of orthographic skill and RAN as compared with average readers. Deficits in RAN and OA might result in weak orthographic representations that lead to problems in automatization and rapid retrieval, thus contributing to failures in reading (Ho et al., 2002). MA appeared to be one of the best skills for distinguishing children with and without dyslexia in several concurrent studies (McBride-Chang et al., 2008; Zhang et al., 2025). It is possible that Chinese children’s inability to differentiate among meanings of homophones and to discriminate morphemes results in their reading failure (Peng et al., 2017).

The role of PA in distinguishing Chinese poor readers from typical readers remains debated. Several studies on children with dyslexia have demonstrated that these children performed more poorly than typically developing children on phonological sensitivity (e.g., Ding et al., 2025). PA was found to play an important role in predicting dyslexia status in Chinese children in some studies (e.g., Song et al., 2020). However, some other studies have found that PA tended to be less powerful in distinguishing children with and without dyslexia in Chinese than in alphabetic orthographies (e.g., Ho et al., 2002, 2004). Therefore, the predictive role of PA in Chinese poor reader status requires further investigation.

Several previous studies have examined the roles of copying skills in distinguishing concurrent poor and typical readers. McBride-Chang et al. (2011a) tested copying skills in third-grade and fourth-grade Chinese children and found that copying tasks showed moderate effect sizes in distinguishing children with and without dyslexia even after RAN, MA, and OA were statistically controlled. Another study also tested the extent to which copying skill uniquely discriminated between those with and without dyslexia in Chinese second graders (Kalindi et al., 2015). However, these two studies both used pure copying tasks. We do not know whether DC, which taps more other specific script-related skills, could be a marker of poor reader status. Some studies have examined that delayed copying directly contributed to Chinese literacy after other variables were statistically controlled; while pure copying had an indirect effect on Chinese literacy through delayed copying (Ye et al., 2022). These studies indicate that the critical role of delayed copying in Chinese literacy makes it a potentially good clinical indicator of literacy proficiency and difficulties. DC is a relatively underexplored variable for predicting poor reader status. It might be a useful indicator for distinguishing those with typical and poor reading because it involves not only orthographic knowledge but also visual-chunking and working memory skills (Pak et al., 2005). A recent study found that DC was a significant predictor of dictation performance of children with dyslexia (Chen et al., 2025). Another study differentiated the subtypes of Chinese dyslexia across three Chinese societies (i.e., Hong Kong, Beijing, and Taipei) and results showed that all four subtypes (i.e., orthographic deficit subtype, phonological deficit subtype, morphological deficit subtype, and global deficit subtype) of dyslexia manifested poorer delayed copying performance as compared to the control group (Cheah et al., 2025). Both studies suggested that DC might play a more prominent role in Chinese dyslexia than previous thought. The present study included DC as one of the possible predictors for poor reader status to fill the gap in this topic.

Previous studies have demonstrated that various cognitive-linguistic skills are reliable factors for distinguishing poor and typical readers when measured concurrently with poor reader status (e.g., Ho et al., 2004). However, these studies did not always yield consistent findings. For example, the role of PA remains less clear in demonstrating variability in Chinese children’s literacy as compared to its association with variability in English word reading (e.g., Ho et al., 2002). In addition, it is unclear how the roles of these predictors might change developmentally.

## Predictors of longitudinal dyslexia/poor reader status in Chinese children

Previous research on early indicators of longitudinal dyslexia or poor reader status in Chinese children have yielded inconsistent findings. On the one hand, some studies have shown that the deficit profiles of poor readers can change with age increase. For example, a study investigated the early cognitive predictors of word reading impairment in Chinese children from ages 3 to 8 found that a deficit in PA appeared at young ages, and a deficit in MA emerged at old ages in poor readers (Lei et al., 2011).

On the other hand, several studies have suggested that most of these cognitive-linguistic skills were longitudinal predictors for Chinese dyslexia or poor reader status (e.g., Wong et al., 2012). For example, a four-year longitudinal study tested a series of cognitive-linguistic skills in Chinese children from ages 4 to 6 and conducted a standardized dyslexia test at age 7 (Ho, 2014). Results of this study revealed that PA and RAN were the best predictors of dyslexic outcome at age 7. Another study by Ho et al. (2011) found that Chinese preschool children who manifested difficulties in word reading at age 6 had histories of deficits in a wide range of cognitive-linguistic skills, including phonological sensitivity and RAN, at ages 4 and 5. MA was also found to be a longitudinal predictor of dyslexia (e.g., McBride-Chang et al. 2011b). McBride-Chang et al. (2011b) conducted a study exploring whether the cognitive-linguistic skills tested at age 5 could distinguish children with and without dyslexia at age 7. They found that MA and RAN were the two significant indicators. Similarly, Tong et al. (2017) followed a group of children with and without dyslexia at ages 6, 7, and 8. Their results suggested that children with dyslexia had deficits in MA and RAN across all three years.

These longitudinal studies suggest that children who are defined as having status of dyslexia or poor reader at later ages show deficits in a set of early cognitive-linguistic skills. However, the cognitive-linguistic skills of children in these studies were mainly measured during the period of kindergartens. We know little about whether the roles of these skills in distinguishing poor and typical readers will be the same in primary school children who start receiving formal literacy instruction. In addition, few of these studies simultaneously included a comprehensive set of cognitive-linguistic skills. The role of DC in predicting longitudinal poor reader status has not yet been explored. Therefore, more longitudinal research is necessary to understand the developmental trajectories of predictors of poor reader status.

## The present study

The present study aimed at examining the possible markers of poor reader status in Chinese children concurrently and longitudinally. In particular, we addressed the predictive roles of different cognitive-linguistic skills and how their roles change with development. The present study sought to extend previous studies in two critical ways. First, a comprehensive set of cognitive-linguistic skills as risk factors were included in this study. These skills contained PA, MA, RAN, OA, and DC. Of particular significance, the present study incorporated DC, which remained understudied, as one of the possible markers of poor reader status. Odegard et al. (2025) suggested that effective policy should integrate dyslexia risk screening within comprehensive early language and literacy protocols, ensuring that all language-based vulnerabilities are monitored from the start rather than siloed into separate processes. Second, Hong Kong Chinese children in first to third grades were recruited to examine the best predictors for distinguishing poor and typical readers concurrently. Furthermore, a subsample of the first graders was followed up for three years to investigate the best indicators for discriminating between poor and typical readers longitudinally. The cross-sectional analyses and the longitudinal comparisons allow a complete trajectory view of predictors for word reading impairment in Chinese children. A longitudinal approach helps understand the developmental trajectories of risk factors of poor reader status. The results will shed light on the identification and intervention for Chinese poor readers and dyslexia. Moreover, the results of the present study could contribute to support for and extend the 2025 IDA dyslexia definition from the perspectives of Chinese orthography.

Based on the features of Chinese script and the findings of the previous studies (e.g., Ho et al., 2002, 2004; Shu et al., 2006), we expected to find that MA, RAN, and OA might be the three most reliable indicators for distinguishing poor and typical readers concurrently and longitudinally. DC might also show the potential to predict Chinese poor reader status both concurrently and longitudinally. The patterns of significant predictors for concurrent and longitudinal poor reader status might be different.

## Method

### Participants

First- to third-grade children were recruited from different Hong Kong primary schools. These schools followed a similar Chinese curriculum released by the Hong Kong Education Bureau, though there may be small differences (such as teaching progress, difficulty of the teaching materials) among schools. Among them, 73.2% of the schools used Cantonese, 11.2% used English, and 2.5% used Mandarin as the medium of instruction. 13.1% of the participants did not report this information. A word reading test (details described under the “Measures” section) was used to identify poor and typical readers among the initial sample of different grades. Children whose word reading scores fell into the bottom 25% of all children in the same grade were identified as poor readers. This is a standard approach that was commonly used to identify poor readers in many previous studies (e.g., Chung et al., 2022; Fong & Ho, 2022; Liu et al., 2010; McBride-Chang et al., 2013; Penney et al., 2005). Typically developing readers as control groups were selected from the remaining children (above the lowest 25%) in the same grade. No other behavioral and mental problems were reported in the participating children.

*Concurrent group.* A total of 358 children participated in the present study. Among them, 211 children were first graders, 86 were second graders, and 61 were third graders. The mean age of the participating children was 7.31 (SD = 0.90). 91.9% of the participants were born in Hong Kong, 2.2% were not, and 5.9% of the participants did not report this information. Participants were from various SES backgrounds. Based on the abovementioned criterion, 94 children (47 male, 47 female) were identified as poor readers, and 94 age and IQ matched children (54 male, 40 female) were selected as typical controls from the remaining children. All children completed measures of cognitive-linguistic skills and word reading ability at the same time.

*Follow-up group*. First-grade children in the concurrent group were invited to complete the reading ability task again one and two years later. A subsample of 109 children completed the reading ability task at all three time points, namely, T1, T2, and T3. The mean age of these children at T1 was 6.64 (SD = 0.42). Children who participated in the study at all time points did not have significant difference in T1 word reading performance with children who only participated in the study at some time points (*t* = -0.56, *p* = .58). We divided these longitudinally followed children into poor and typical readers at different time points according to their word reading performance each time. Therefore, 28 of the 109 children were identified as poor readers at T1 and 29 as poor readers at T2 and T3. Matched typical reader group consisted of 29 children whose word reading scores did not fall into the bottom 25% at any of the three time points. The persistent rate of reading difficulty from T1 to T2 was 85.7%, and this rate from T1 to T3 was 67.9%. The word reading performance of children who were identified as poor readers at different time points located below or at the average level in all three years. This indicates that the poor reading status tends to be persistent in primary school children.

Overall, the present study included a concurrent group and a follow-up group. Children in the concurrent group completed measures of cognitive-linguistic skills and reading ability concurrently. For the follow-up group, measures of cognitive-linguistic skills and reading ability were conducted in the first year (T1), and the reading ability was tested again one year (T2) and two years (T3) later.

## Measures

*Nonverbal IQ.* Items from sections A to C of Raven’s Progressive Matrices (Raven et al., 1976) were used to estimate nonverbal IQ. Participants were shown a matrix with a missing part and were asked to select a figure to best fit the missing part from six to eight figures for each item. There were 36 items in this test, and one point was awarded for each correct answer.

*PA.* PA task consisted of two sections: syllable deletion and onset deletion. This task was selected because the primary phonological units (grains) of Chinese are syllables, onsets, and rimes. Chinese readers attend to these units to process phonological information during word reading. Therefore, syllable and onset deletion tasks could be used to measure PA of Chinese more effectively than tasks that focus on other phonological grain-size units. This is different from alphabetic orthographies, in which phoneme-size units are primarily used in phonological processing. Different grain-size units used in manipulating phonological information might result in different predictive roles of PA in poor reader status across orthographies. This task worked similarly to the style of the Comprehensive Test of Phonological Processing (CTOPP, Wagner et al., 1999). Items were arranged with increasing difficulty, and children started the test with the level according to their grades. For the syllable deletion section, experimenters presented a three-syllable word orally each time. Children needed to repeat the word with one syllable deleted. For example, the three-syllable word /kun1 mek1 paau6/ without saying the syllable /paau6/ would be /kun1 mek1/. There were 19 items in the syllable deletion section. For the onset deletion section, experimenters presented a one- to three-syllable word orally each time. Children needed to repeat the word with the onsets in each syllable deleted. For example, the two-syllable word /pe2 tat6/ without saying the onset (i.e., p and t) in each syllable would be /e2 at6/. There were 22 items in the onset deletion section. One point was given for each correct answer. Testing stopped if children answered five or more items incorrectly within the same level. The maximum score of this test was 41.

*MA.* MA task consisted of two sections: morphological construction and compounding production. Children began the task from the level corresponding to their grades. In this test, children needed to orally create a new word for an object or concept. In the morphological construction section, children were given a scenario before creating the new word for each item. For example, “The webs made by spiders are called spider webs. What should we call the webs made by ants?” The answer should be “ant webs.” There were 27 items in this section. In the compounding production section, children were not presented with scenarios anymore. For example, “What should we call a lion that has long arms?” The answer should be “long-armed lion.” There were 19 items in this section. A stopping rule was applied when children answered four or more items incorrectly within the same level. One point was awarded for each correct answer. The maximum score of this test was 46.

*RAN.* A rapid digit naming task was used to assess the RAN skill. This task used a number list consisting of eight rows. The numbers 2, 4, 6, 7, and 9 were contained in each row in random orders. Children needed to name the digits from left to right row by row as quickly and accurately as possible. The task was conducted twice. The average naming time across the two trials was calculated.

*OA.* A character decision task was used to assess OA. Chinese characters comprise various radicals/components. Radicals/components are the orthographic sub-units of Chinese, and they are the primary grains that Chinese readers use in processing orthographic information during reading acquisition. This task contained 30 rare Chinese characters and 30 noncharacters. Each character comprised of two components arranged in a left-right structure. Rare characters were real Chinese characters of low frequencies at the primary school levels. Noncharacters were constructed by combining a semantic radical and a phonetic radical in their illegal positions or by combining two semantic radicals or phonetic radicals that violate the conventional rules of Chinese characters. Children were required to identify whether each character was a real character within one minute. One point was awarded to each correct answer. The possible maximum score was 60.

*DC.* Similar tasks have been used in previous studies (e.g., Anderson et al., 2013). In this test, children were presented with a character for 2 seconds on the computer screen each time. They were required to remember the character and rewrite it after it disappeared. Characters used in this test were rare characters with low use frequency for children at this age. Rare characters were chosen in this task to ensure children could not retrieve a pre-formed lexical representation and must instead rely on visual-chunking, working memory, and component-level orthographic processing skills. Each character comprised 2 to 4 components (i.e., radicals and logographemes). For example, the character “侫” composed of three components: “亻”, “亡”, and “女”. Components contained in the rare characters might be common in Chinese, and children may be familiar with some of the components. Children could use their knowledge of the familiar components for chunking some of the strokes so that they could remember as many strokes as possible. However, the unfamiliar combinations of the components in the rare characters ensured that the task taps the processing of visual chunking rather than lexical memory. This task was different from the OA task, which measured children’s sensitivity to legitimate structures of Chinese characters. In this test, two points were awarded to each correctly written component. One point was given if a minor error was observed within a component (e.g., a stroke was missed within the component). There were 15 characters containing 41 components totally in this test. The maximum score of this test was 82.

*Word Reading.* This test used a Chinese word list consisting of 150 two-character words. Children were required to read aloud the words one by one. These words were arranged in the order of increasing difficulty. All words were selected from Hong Kong kindergarten and primary school textbooks. One point was awarded to each correctly read word. The test stopped when children read 15 consecutive words incorrectly. The maximum possible score for this test was 150.

### Data analysis

A series of ANCOVAs were conducted to compare the performances of different cognitive-linguistic skills between poor and typical readers in the concurrent group. Logistic regression analysis was conducted to find predictors that best distinguish poor readers from typical readers in the concurrent group. For the follow-up group with longitudinal data, the same logistic regression analyses were performed at three time points individually to investigate early predictors for later poor reader status. All predictors were put together into the same model during the analyses. In the concurrent group, one first-grade poor reader did not complete the RAN task, and three third-grade poor readers did not perform the OA task. Therefore, individual grade mean of the respective measure were used to impute the missing data.

## Results

### Differences between poor and typical readers in the concurrent group

Table 1 presents means, standard deviations, and separate *F* tests for all measures administered to the poor and typical reader groups. There were no significant differences in age (*t* = 0.18, *p* = .86) and IQ (*t* = 1.62, *p* = .11) between the two groups. With age and IQ controlled, ANCOVA analyses results showed that the poor reader group performed significantly worse on all measured cognitive-linguistic skills than the typical reader group.

**Table 1:** Reliabilities, means, standard deviations, and *F*s of all tasks administered to poor and typical readers in the concurrent group

Variable	Reliability	Poor readers (*n* = 94)		Typical readers (*n* = 94)		F (1, 184)
		M	SD	M	SD	
Age		7.24	0.89	7.26	0.95	
IQ	0.87	21.90	5.97	23.34	6.19	
Word reading	0.99	30.43	24.45	76.14	28.95	283.82^***^
Phonological awareness	0.95	23.84	8.81	29.89	7.96	25.25^***^
Morphological awareness	0.89	17.09	5.62	22.97	5.44	66.07^***^
Orthographic awareness	0.90	19.58	9.15	22.76	7.42	7.98^**^
Delayed copying	0.93	29.14	14.57	41.70	16.48	30.19^***^
RAN	0.86	26.42	7.24	21.45	7.07	24.80^***^

Test–retest reliability was computed for the RAN task; Cronbach’s *α* was reported for the remaining tasks. ^**^ *p* < .01, ^***^ *p* < .001

### Correlations between cognitive-linguistic skills and word reading ability in the concurrent group

Table 2 presents the correlation coefficients for all measured cognitive-linguistic skills and word reading with age and IQ controlled. Results showed that all the measured cognitive-linguistic skills correlated significantly with word reading.

**Table 2:** Correlations between cognitive-linguistic skills and word reading with age and IQ controlled in the concurrent group (*n* = 188)

Variable	1	2	3	4	5	6
1. Word reading	1.00					
2. Phonological awareness	0.43^***^	1.00				
3. Morphological awareness	0.61^***^	0.45^***^	1.00			
4. Orthographic awareness	0.27^***^	0.07	0.13	1.00		
5. Delayed copying	0.36^***^	0.18^*^	0.37^***^	0.10	1.00	
6. RAN	− 0.40^***^	− 0.33^***^	− 0.27^***^	− 0.15^*^	− 0.19^*^	1.00

^*^ *p* < .05, ^***^ *p* < .001

### Distinguishing poor readers from typical readers in the concurrent group

Logistic regression analysis was carried out to examine the best predictors to distinguish poor readers and typical readers concurrently. The five measured cognitive-linguistic skills were entered simultaneously in the logistic regression analysis with Wald’s forward stepwise selection. Age and IQ were controlled in the model. MA (*χ*^*2*^ (1, *N* = 188) = 22.77, *p* < .001) and DC (*χ*^*2*^ (1, *N* = 188) = 8.91, *p* < .01) were significant predictors to distinguish poor and typical readers concurrently. Table 3 shows the accuracy of predicting children’s status as good or poor readers. With the two predictors, the overall correct prediction rate was 72.3%. The accuracy rates were 72.3% for both poor and typical readers.

**Table 3:** Prediction of children’s status (concurrent group)

Observed	Predicted (*n*)		
	Typical readers	Poor readers	Prediction rate
Typical readers	68	26	72.3%
Poor readers	26	68	72.3%
Overall prediction rate			72.3%

### Distinguishing poor readers from typical readers longitudinally in the follow-up group

We then performed logistic regression analyses with Wald’s forward stepwise selection to examine the best predictors to distinguish poor readers from typical readers at three time points separately. Results showed that MA (*χ*^*2*^ (1, *N* = 57) = 8.71, *p* < .01), OA (*χ*^*2*^ (1, *N* = 57) = 4.94, *p* < .05), and RAN (*χ*^*2*^ (1, *N* = 57) = 5.37, *p* < .05) were significant predictors to distinguish poor readers and typical readers at T1. The overall correct prediction rate was 77.2%. At T2, MA (*χ*^*2*^ (1, *N* = 58) = 4.92, *p* < .05), OA (*χ*^*2*^ (1, *N* = 58) = 7.09, *p* < .01), DC (*χ*^*2*^ (1, *N* = 58) = 4.17, *p* < .05), and RAN (*χ*^*2*^ (1, *N* = 58) = 4.58, *p* < .05) measured at T1 were significant predictors in the model. The overall correct prediction rate was 81.0% at T2. At T3, MA (*χ*^*2*^ (1, *N* = 58) = 4.30, *p* < .05) and OA (*χ*^*2*^ (1, *N* = 58) = 5.51, *p* < .05) measured at T1 were significant predictors. RAN also reached a marginally significant level (*χ*^*2*^ (1, *N* = 58) = 3.70, *p* = .05). The overall correct prediction rate was 72.4% at T3. Table 4 shows the prediction accuracy of children’s status at different time points. These results indicate that early performances on specific cognitive-linguistic skills are useful in assessing the presence of future poor readers among Chinese primary school children. Early MA, OA, and RAN have strong associations with future poor reader status in Chinese children.

**Table 4:** Prediction of children’s status at T1, T2, and T3 (follow-up group)

	Predicted typical readers			Predicted poor readers			Prediction rate		
	T1	T2	T3	T1	T2	T3	T1	T2	T3
Observed typical readers	22	23	22	7	6	7	75.9%	79.3%	75.9%
Observed poor readers	6	5	9	22	24	20	78.6%	82.8%	69.0%
Overall prediction rate							77.2%	81.0%	72.4%

Results of the final models for all logistic regression analyses conducted in the concurrent group and the follow-up group are summarized in Table 5.

**Table 5:** Results of the final model of logistic regression in different groups: B (SE)

Variables	Concurrent group	Follow-up group		
		T1	T2	T3
Phonological awareness (T1)	-	-	-	-
Morphological awareness (T1)	− 0.18 (0.04) ^***^	− 0.34 (0.12) ^**^	− 0.26 (0.12) ^*^	− 0.20 (0.10) ^*^
Orthographic awareness (T1)	-	− 0.16 (0.07) ^*^	− 0.22 (0.08) ^**^	− 0.17 (0.07) ^*^
Delayed copying (T1)	− 0.03 (0.01) ^**^	-	− 0.06 (0.03) ^*^	-
RAN (T1)	-	0.13 (0.06) ^*^	0.12 (0.06) ^*^	0.10 (0.05) ^a^

^*^ *p* < .05, ^**^ *p* < .01, ^***^ *p* < .001, ^a^ *p* = .05. Poor reader was coded as 1 and typical reader was coded as 0. “-” represents the variable was not included in the final model

## Discussion

Examining the predictors of concurrent and longitudinal poor reader status in Hong Kong Chinese primary school children yielded several notable results in the present study. For the concurrent results, MA and DC were the best indicators to distinguish poor and typical readers in the concurrent group. For the longitudinal results, MA, OA, and RAN measured in the first year were the significant predictors of poor reader status one and two years later in the follow-up group. PA failed to distinguish poor and typical readers both concurrently and longitudinally. These findings are discussed in relation to previous work.

### Similar patterns of predictors for concurrent and longitudinal poor reader status

The contribution of MA in distinguishing poor and typical readers was the most stable concurrently and longitudinally. This result is consistent with many previous studies, which have examined that MA is highly relevant for Chinese word reading acquisition across different stages (e.g., Song et al., 2020; Tong et al., 2017). The 2025 IDA dyslexia definition specially proposed morphological processing deficit as one of the common difficulties in dyslexia (IDA, 2025). The unique importance of MA in Chinese word reading development has been well documented (e.g., Liu et al., 2017). The ability to recognize morphemes and manipulate the morphemic structure of words is essential to achieving reading proficiency in Chinese (Liao et al., 2020). Some researchers suggested that knowing the way to combine individual morphemes into multi-morphemic words helps children to make educational guesses for the meaning of words and thus facilitate word reading (Zhou et al., 2014). The properties of the Chinese writing system might determine MA as the specific marker of poor reader status. A Chinese character represents a morpheme itself. Morpheme-size units and character-morpheme correspondence play critical roles in Chinese word reading (Catts et al., 2026). Moreover, there is extensive word compounding in Chinese, which makes MA important for distinguishing word meanings in Chinese. Mastering the meaning of morphemes may also greatly assist children in distinguishing different homophones (Liu & McBride-Chang, 2010). In contrast, these features are less prominent in alphabetic writing system, where written phonemes map onto graphemes. MA appeared to be the most notable skill to distinguish poor and typical readers in the present study, while fluency training appeared to be the most effective type of intervention for children’s literacy skills in Ruan et al. (2024)’s meta-analysis. This difference might result from some reasons. First, the number of intervention studies that only focused on training fluency is very limited. As noted in Ruan et al. (2024), the small number of included studies might result in some bias for the fluency intervention effect. Second, the efficacy of the intervention could be influenced by many factors, such as the settings of the intervention. Ruan et al. (2024)’s study did not control for all variations during the meta-regression analyses due to the limited number of included studies. In contrast, the present study controlled other cognitive-linguistic skills during the analyses. Therefore, the results might be different because of various controlled variables. Although this small difference, the present study still found the importance of RAN in predicting longitudinal poor reader status, and Ruan et al. (2024)’s study also revealed the significant effect of morphological intervention. Both studies consistently examined the important role of MA, fluency, and OA in Chinese literacy.

In contrast to these consistent findings in relation to MA, PA did not significantly predict concurrent and longitudinal poor reader status. PA was affected in poor readers; however, it does not emerge as a significant predictor in Chinese when considering other skills together. This result might be attributed to the characteristics of Chinese orthography. According to the 2025 IDA dyslexia definition, deficit in phonological processing is a common but not universal difficulties in dyslexia (IDA, 2025). The underlying difficulties in cognitive-linguistic skills of dyslexia might vary depending on the orthography. Chinese and alphabetic orthographies vary in the grain size of lexical representations. Morphemes/characters are the lexical representations of Chinese, and phonemes are often the core lexical representations of alphabetic orthographies. According to the psycholinguistic grain size theory, different orthographies use various grain-size units to decode words (Ziegler & Goswami, 2005). The role of cognitive-linguistic skills in predicting poor reader status might be different across orthographies if they rely on different grain-size units to decode words. Chinese characters provide limited reliable phonological information. Readers may have to rely on the level of semantic cues rather than phonological cues in print to learn to read (McBride, 2016). Previous studies have found that few Chinese children with dyslexia exhibited a deficit in PA (e.g., Ho et al., 2002). Penney et al. (2005) also revealed that good and poor readers did not differ on PA. Therefore, PA might be less important for Chinese literacy development, at least in certain contexts, as compared to its utility for reading in alphabetic languages. Our result is in line with many previous studies, which found that PA was not a significant predictor for distinguishing good and poor readers in Chinese (e.g., Kalindi et al., 2015). Some researchers have suggested that PA is likely an early contributor to reading development in very young children but may not emerge as a unique correlate of Chinese reading later (e.g., Ho, 2014). Children in the present study were in primary school. This might partly explain why we did not find the predictive role of PA in Chinese poor reader status. However, several studies on children in Mainland China found that PA played a role in predicting dyslexic status (e.g., Song et al., 2020). The inconsistent finding might be because we controlled other cognitive-linguistic skills in our study. Hence, the role of PA might vanish after considering other more important predictors. Moreover, unlike in Mainland China, there is no phonological coding system used to facilitate character teaching in Hong Kong. Therefore, PA might be relatively limited in predicting poor reader status in Hong Kong Chinese children, particularly beyond their earliest years of literacy learning. This result suggests that the features of writing system and the contexts of the region are important factors that needed to be considered when identifying poor readers or children with dyslexia.

It should be noted that the correlation between MA and PA was significant in the present study, indicating that MA and PA might share variance with each other. One reason for this finding is that a Chinese character typically represents both a morpheme and a syllable simultaneously. Therefore, the PA task that requires manipulating syllable-level units might have some common processing with the MA task that requires manipulating morphemic units. However, one main distinction between the PA and MA tasks in the present study is the extent to which meaning plays a role in the task. Specifically, the PA task required children to repeat aloud a three-syllable word with one syllable deleted. The three-syllable strings were meaningless, and children only focused on manipulating the phonological information they were orally presented with. In contrast, the MA task heavily emphasized children’s ability in processing meaning related information. In the MA task, children needed to orally create a new word for an object or concept based on given scenarios. Hence, children were required to understand the meaning or at least guess the meaning of the given morphemes and then combine them into Chinese compounds. Although the MA task might activate syllable-level phonological processing, the core of this task is still to assess children’s ability to manipulate the basic meaning units in Chinese. The significant correlation between PA and MA is very common in reading research, even in alphabetic languages (e.g., Lee et al., 2023). Hence, the consistent result of MA and the null finding of PA in the present study are likely to be attributable particularly to the specific features of Chinese orthography rather than the shared variance between PA and MA tasks.

DC showed good potential as a predictor of poor reader status both concurrently and longitudinally. This result is consistent with a recent study that found different subtypes of Chinese dyslexia all manifested a delayed copying deficit (Cheah et al., 2025). The role of DC has been relatively understudied thus far in both Chinese and other writing systems. DC might be potentially crucial because learning Chinese requires attention to the characters’ details during visual motor integration and relies on visual-spatial skills (Kalindi et al., 2015). DC requires children to temporarily hold stroke patterns of characters in mind and retrieve them during the process of reproducing characters by motor execution. Therefore, DC involves multiple skills, including visual-orthographic knowledge, visual-chunking skill, orthographic working memory, and handwriting skills (Ruan et al., 2023). Among these multiple skills, orthographic working memory, distinguished from orthographic knowledge, might be the most crucial one. A study examined that the role of orthographic knowledge diminished when considering the contributions of DC to word spelling simultaneously (Mo et al., 2018). This situation might also apply to word reading. Besides the logistic regression results, the descriptive and correlational results revealed that poor readers manifested worse DC skills than typical readers, and DC was significantly correlated with word reading. Besides the Chinese writing system, a few studies in other language systems have examined that DC is important for literacy skills (e.g., Cho, 2021; Shin et al., 2026). In addition, studies on Chinese-English bilingual children also examined that English delayed copying was a unique correlate of English word reading and spelling after statistically controlling for other literacy- related skills (Lo et al., 2018; Ruan et al., 2023). In this case, the role of DC in predicting concurrent and longitudinal poor reader status is worth further establishing in future research.

### Different patterns of predictors for concurrent and longitudinal poor reader status

The patterns of predictors for concurrent and longitudinal poor reader status have two main differences. First, the longitudinal analysis identified a larger number of predictors for distinguishing typical and poor readers than the concurrent analysis. This result might indicate that some early measured cognitive-linguistic skills do not have an immediate role but their predictive role become prominent with time development. Some studies have examined that the deficit profiles of poor readers would change with age increases (e.g., Lei et al., 2011). Second, RAN and OA were not markers of concurrent poor reader status but were significant predictors of longitudinal poor reader status.

RAN and OA measured at T1 were the two most stable early predictors of longitudinal reading status across three years besides MA in the follow-up group. Research on exploring early correlates of later dyslexia in older primary school children also identified RAN, MA, and OA as the most robust markers (e.g., Ho et al., 2002, 2004; Shu et al., 2006). RAN has been found to strongly correlate with reading difficulty across orthographies (e.g., Ding et al., 2025). Some researchers have argued that reading process requires the linkage between visual and phonological information that similar to the procedure of RAN tasks (e.g., McBride-Chang et al., 2013). Therefore, RAN plays an important role in reading ability. RAN taps not only the skills involving extracting orthographic patterns and corresponding sounds automatically but also the degree of automaticity in lexical retrieval (e.g., Ho, 2014). The deficit in RAN might indicate the disruption of the automatic processes involved in the extraction of orthographic patterns, thus causes reading failure ( Peng et al., 2017). RAN as a basic cognitive-linguistic skill required by reading acquisition could be a significant predictor of later poor reader status. However, other skills might compensate the effect of RAN in older children. Therefore, RAN was not a significant marker for poor reader status in the concurrent group that contained older children in the present study.

OA measured at T1 consistently predicted poor reader status at T2 and T3 in the follow-up group. However, OA was not a significant indicator for poor reader status in the concurrent group. The results indicate that the OA deficit in young ages was an essential indicator of subsequent poor reader status. Previous research has shown that a deficiency in OA is one of the most dominant difficulties in Hong Kong children with dyslexia (e.g., Ho et al., 2004). The deficit in OA might indicate the inability to recognize different aspects contained in characters and thus resulting in reading failure. However, the role of OA in the present study seems not that prominent in older children. This might be because the OA measured in the present study is only one of several aspects of orthographic knowledge. The present study used a lexical decision task that tested the conventional rules of forming Chinese characters to measure OA. Orthographic knowledge involves recognizing and processing character configuration and structure, radical information, and positional and functional aspects of character components (e.g., Ho, 2014). Therefore, it is possible that different aspects of orthographic knowledge play different roles in various developmental stages. Future studies can explore different aspects of orthographic knowledge in predicting poor reader status in older children more in depth.

### Limitations, future directions, and implications

The present study has several limitations. First, although we have included a comprehensive set of cognitive-linguistic predictors for Chinese poor reader status, other risk factors still need to be considered when exploring the markers of poor readers. For example, the role of oral language in predicting poor reader status should be also examined in future research. The 2025 IDA dyslexia definition reinforces the early oral language weaknesses in dyslexia and researchers have suggested that oral language assessment could be incorporated into the screening tool for dyslexia (Odegard et al., 2025). Second, the sample size of grades 2 and 3 children in the present study is relatively small. In addition, our poor reader sample did not receive formal diagnoses of dyslexia. It is not easy to find children with clinically diagnosed dyslexia in such a narrow age range within limited participating schools. However, the present study does shed light on the identification of Chinese dyslexia due to the similar judgment criterion (i.e., difficulty in word reading) for poor readers. Future studies should include a larger sample size of children and further examine the concurrent and longitudinal indicators for poor reader status. Third, a spelling measure was not included in the present study. In the present study, only word reading was used as the outcome variable of poor reader. According to the 2025 IDA dyslexia definition, difficulties in word spelling often accompany word-reading difficulties in children with dyslexia. Therefore, a complete exploration of the full scope of the IDA dyslexia definition should also involve the spelling dimension. In alphabetic orthographies, spelling refers to the encoding process of grapheme-to-phoneme correspondences (Catts et al., 2026). In Chinese, there are no grapheme-to-phoneme correspondences; instead, dictation is usually treated as the functional analog of spelling. A few studies have shown that cognitive-linguistic skills similar to those tested in the present study were linked significantly with dictation performance in Chinese dyslexia. For example, Chen et al. (2025) revealed that delayed copying was a significant predictor of dictation in Chinese dyslexia, and Liu et al. (2024) found that orthographic awareness and dictation subtests in a dyslexic screen tool were significantly correlated. These limited studies support the idea that the cognitive-linguistic skills in this study, such as DC and OA, might also be significant predictors of the spelling performance of Chinese poor readers. As indicated in the Catts et al. (2026) article, written language systems differ in numerous ways that affect word reading and spelling. Hence, the findings in the present study might also reflect the potential roles of measured cognitive-linguistic skills in dictation/spelling difficulties in poor readers under the Chinese orthography context. Research on the nature of spelling-equivalent deficits in Chinese dyslexia is still relatively scarce. Future studies should include a dictation measure as an outcome variable alongside word reading. In addition, population-level research including the subtyping studies of dyslexia should also be considered in the future. These would be helpful for applying the full scope of the latest IDA definition to Chinese orthography.

Nevertheless, the present study has both theoretical and practical implications. Theoretically speaking, looking into predictors for concurrent and longitudinal poor reader status in Chinese children helps build universal reading theories across different orthographies and understand the specific features of Chinese reading failure. For example, we examined different roles of cognitive-linguistic skills in explaining the reading variety in various developmental stages. These findings support some theories that highlight the importance of these skills on reading acquisition across different languages and writing systems (e.g., the triangle model developed by Seidenberg & McClelland, 1989). In addition, we found that PA was specifically not a significant indicator for Chinese word reading failure when included with other skills focused on morphological and orthographic skills. However, studies in alphabetic languages have particularly emphasized the important role of PA in reading development (e.g., Yeong et al., 2014). This finding indicates that different languages and scripts may yield some specific manifestations in reading failure. The grain size of units used in decoding words varies in different orthographies, which might be the main reason for this finding (Ziegler & Goswami, 2005). The results of the present study not only support for the 2025 IDA dyslexia definition that specifically noted morphological processing deficit in dyslexia (IDA, 2025), but also extend the definition to orthographic processing. The contexts of different writing systems should be considered when defining dyslexia. Orthographic processing might be an important risk factor of dyslexia in morpho-syllabic orthographies, such as Chinese. Practically speaking, understanding the predictors of poor reader status can improve the accuracy of the early assessment of dyslexia. For example, tools used to screen dyslexia can consider including the measurements of the examined significant predictors. In addition to giving a traditional reading screening only focused on word recognition in many Chinese dyslexia or poor reader studies (e.g., Song et al., 2020), the accuracy of diagnosis might be improved by conducting the screening of cognitive-linguistic skills with salient predictive roles for poor reader status. Based on our findings, an adequate test battery to distinguish Chinese children with and without word reading difficulty might at least contain tasks of MA, DC, orthographic knowledge, and fluency in addition to word reading. Furthermore, knowing the concurrent and longitudinal roles of predictors in contributing to poor reader status can help develop effective curricula and interventions for poor readers at different stages. For example, training focusing on improving MA might effectively ameliorate severe word reading problems in Chinese poor readers. Teachers should integrate some techniques focusing on training MA in the teaching of Chinese word reading. In addition, OA and RAN skills should be emphasized in teaching reading to young children in schools. In summary, early identification of children at risk of the poor reader can facilitate better poor reading risk management and promote effective interventions. With early identification of potential poor readers, effective intervention can be provided to help children at risk as early as possible.

## Data Availability

The data that support the findings of this study is available upon reasonable request from the corresponding author.
